# The role of the cGAS-STING pathway in chronic pulmonary inflammatory diseases

**DOI:** 10.3389/fmed.2024.1436091

**Published:** 2024-10-30

**Authors:** Mengxiang Tian, Fengyuan Li, Haiping Pei, Xiaoling Liu, Hongyun Nie

**Affiliations:** ^1^Department of General Surgery, Xiangya Hospital, Central South University, Changsha, China; ^2^National Clinical Research Center for Geriatric Disorders, Xiangya Hospital, Central South University, Changsha, China; ^3^School of Medicine, Nanjing University of Chinese Medicine, Nanjing, China; ^4^Department of General Surgery, The 306th Hospital of PLA-Peking University Teaching Hospital, Beijing, China

**Keywords:** cGAS-STING, pulmonary fibrosis, chronic obstructive pulmonary disease, asthma, STING inhibitors, nanomaterial application

## Abstract

The innate immune system plays a vital role in the inflammatory process, serving as a crucial mechanism for the body to respond to infection, cellular stress, and tissue damage. The cGAS-STING signaling pathway is pivotal in the onset and progression of various autoimmune diseases and chronic inflammation. By recognizing cytoplasmic DNA, this pathway initiates and regulates inflammation and antiviral responses within the innate immune system. Consequently, the regulation of the cGAS-STING pathway has become a prominent area of interest in the treatment of many diseases. Chronic inflammatory lung diseases, such as chronic obstructive pulmonary disease (COPD), asthma, and pulmonary fibrosis, are characterized by persistent or recurrent lung inflammation and tissue damage, leading to diminished respiratory function. This paper explores the mechanism of action of the cGAS-STING signaling pathway in these diseases, examines the development of STING inhibitors and nanomaterial applications, and discusses the potential clinical application prospects of targeting the cGAS-STING pathway in chronic inflammatory lung diseases.

## 1 Backgrounds

Chronic pulmonary inflammatory diseases are a group of conditions characterized by chronic inflammation and progressive lung function impairment, primarily including pulmonary fibrosis, asthma, and chronic obstructive pulmonary disease (COPD) (1, 2). Although pulmonary fibrosis is uncommon, it has a poor prognosis. Despite the ability of antifibrotic drugs to slow disease progression, there is no cure, and patients often succumb to respiratory failure (3, 4). Asthma, one of the most common chronic respiratory diseases worldwide, requires long-term and continuous treatment (5, 6). COPD, the fourth leading cause of death globally, affects over 200 million people and is often accompanied by multiple comorbidities, necessitating comprehensive treatment and long-term management, resulting in high medical costs (7). These diseases impose a significant medical and socioeconomic burden, highlighting the urgent need for new therapeutic approaches to optimize treatment regimens, alleviate patient burden, and improve prognosis and quality of life.

The innate immune system is the host’s first line of defense against infection, capable of rapidly responding to pathogen invasion. It comprises a variety of cell types and molecules, including macrophages, dendritic cells, neutrophils, and natural killer cells (8). This system primarily protects the host through pattern recognition receptors (PRRs), which are present on the surface or inside innate immune cells and recognize pathogen-associated molecular patterns (PAMPs). PAMPs are conserved and invariant structures in microorganisms, such as lipopolysaccharide (LPS) in bacteria and double-stranded RNA in viruses (9). When PRRs recognize PAMPs, they initiate a series of signaling pathways that ultimately trigger an immune response. These signaling pathways typically activate transcription factors such as nuclear factor kappa B (NF-κB), leading to the production and release of inflammatory factors, chemokines, and antimicrobial proteins (10, 11). These molecules recruit and activate more immune cells to the site of infection, where they engulf and destroy pathogens while also initiating an adaptive immune response, laying the foundation for a more durable and specific immune defense.

The cyclic guanylate-adenylate synthase (cGAS)–stimulator of interferon genes (STING) pathway plays a crucial role in the innate immune system. However, abnormal activation of this pathway can lead to excessive inflammatory responses. For example, in autoimmune diseases such as systemic lupus erythematosus and Aicardi-Goutieres syndrome (AGS), patients produce a large amount of their own DNA antibodies, leading to the activation of the cGAS-STING signaling pathway and triggering an inflammatory response (12, 13). Additionally, inflammation caused by overactivation of the cGAS-STING pathway has been observed in some chronic inflammatory diseases (14).

Understanding and controlling lung inflammation is vital for the prevention and treatment of chronic respiratory diseases, thereby reducing their global health burden (15). Studies have found that the cGAS-STING signaling pathway plays an important role in chronic inflammatory lung diseases. Abnormal activation of this pathway can lead to chronic inflammation and tissue damage, which can contribute to or exacerbate various chronic inflammatory lung diseases (16, 17). This paper describes the role of the cGAS-STING pathway in chronic inflammatory lung diseases, the application of STING-targeting drugs and nanomaterials, and evaluates the potential of the cGAS-STING signaling pathway as a new therapeutic approach for inflammatory lung diseases.

## 2 The activation mechanism of cGAS-STING signaling pathway

### 2.1 The generation of cytosolic DNA and activation of cGAS

The innate immune system is the body’s first line of defense against pathogen invasion, with its primary function being the rapid identification and response to infections. The cGAS-STING pathway is a crucial DNA recognition and signal transduction mechanism within the innate immune system, playing a significant role in detecting intracellular DNA and initiating immune responses (18).

Under normal circumstances, DNA, which stores our genetic information, is confined to the nucleus and mitochondria of cells. However, when DNA is present in the cytoplasm due to cell damage, apoptosis, or other reasons, it is perceived by cells as a danger signal. This mispositioned self-DNA can trigger endogenous inflammation by activating various innate immune receptors, leading to an inflammatory response in the host (19–21). Cytoplasmic pattern recognition receptors (PRRs), such as cyclic guanylate-adenylate synthase (cGAS), recognize and bind to cytoplasmic DNA. Upon recognizing DNA, cGAS catalyzes the production of cyclic guanylate-adenylate monophosphate (cGAMP), a second messenger that activates the STING (stimulator of interferon genes) pathway, ultimately triggering downstream inflammatory responses (22, 23).

cGAS acts as a DNA sensor in the cytoplasm, recognizing both exogenous DNA (such as viral DNA) and endogenous DNA (such as mitochondrial DNA). When cGAS binds to DNA, it catalyzes the synthesis of cGAMP. As a second messenger, cGAMP directly binds to STING, activating it (24).

### 2.2 The activation of STING and downstream signaling pathways

STING is an essential protein located on the membrane of the endoplasmic reticulum (ER), existing as a dimer (two STING molecules form a functional unit). The two cytoplasmic domains of STING form a V-shaped binding pocket facing the cytosol for binding cGAMP ligands. When STING binds to cGAMP, cGAMP tightly binds to the ligand-binding domain in STING’s V-shaped binding pocket, causing a 180° rotation of the STING ligand-binding domain relative to the transmembrane domain. This rotation brings STING’s C-terminal domains closer together, activating downstream signal transduction (25–27). Activation of STING’s C-terminal domain leads to the recruitment and activation of TBK1 (TANK-binding kinase 1), which phosphorylates IRF3 (interferon regulatory factor 3). Phosphorylated IRF3 dimerizes and enters the nucleus, promoting the expression of type I interferons (such as IFN-*β*) and other inflammatory factors. Additionally, STING activation is not limited to TBK1 and IRF3; it can also activate NF-κB via the inhibitor of kappa B kinase (IKK) complex. Activated STING interacts with the IKK complex, resulting in the phosphorylation and degradation of IκB, releasing NF-κB. Activated NF-κB then enters the nucleus and promotes the expression of multiple chemokines (28).

**Figure 1:**
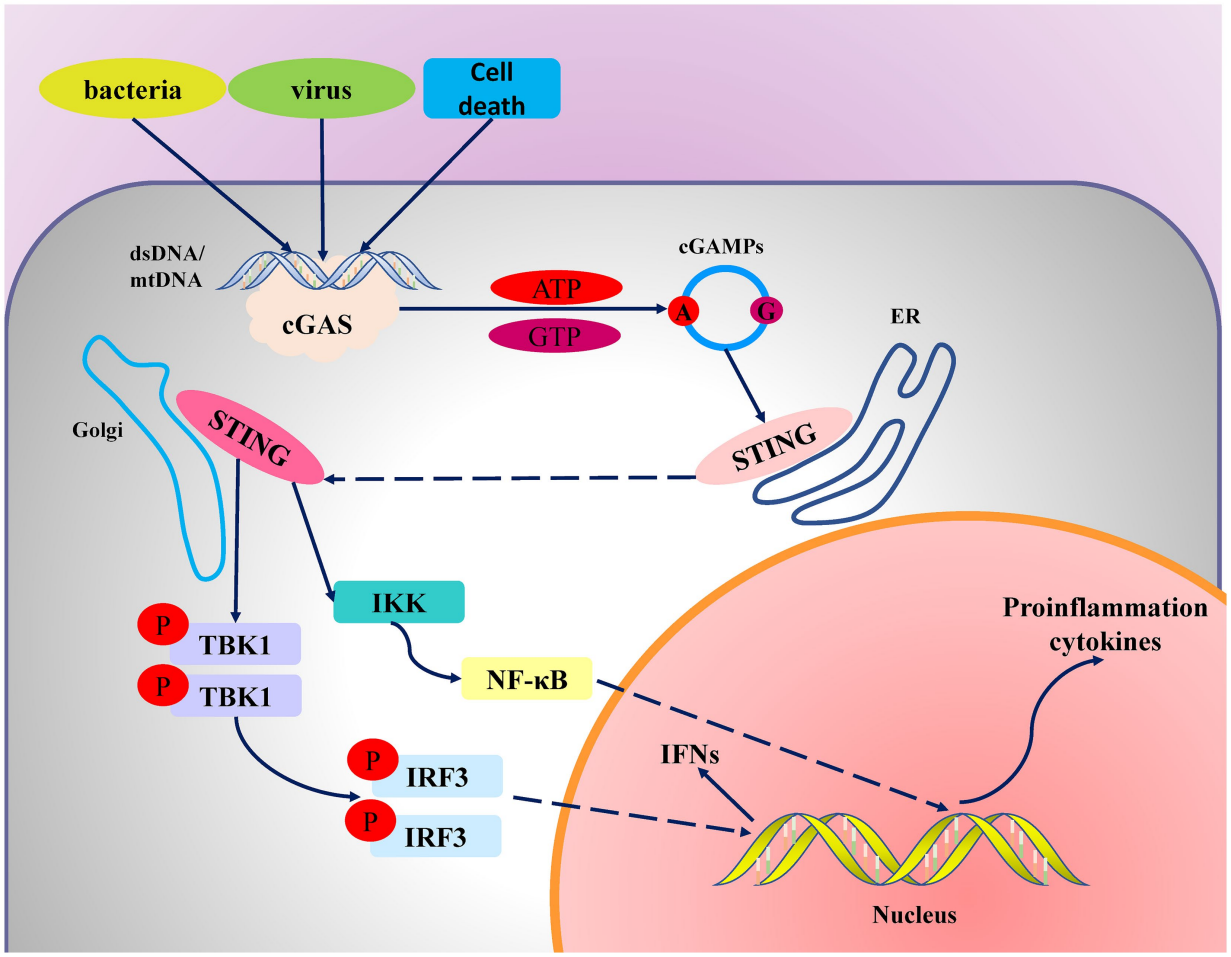
Mechanism diagram of the cGAS-STING signaling pathway. When viral or bacterial infections or genomic instability occur in cells, endogenous or exogenous DNA enters the cytoplasm. cGAS binds to the DNA, undergoes a conformational change, and catalyzes the synthesis of cyclic GMP-AMP (cGAMP) from GTP and ATP. cGAMP then binds to STING, causing STING to translocate from the endoplasmic reticulum to the Golgi apparatus, where it activates the downstream signaling molecule TBK1 (tank binding kinase 1). TBK1 subsequently phosphorylates and activates IRF3 (interferon regulatory factor 3). The activated IRF3 enters the nucleus and initiates the release of type I interferons. Additionally, STING activates the IKK (I-κB kinase) complex, further activating the NF-κB pathway to promote the expression of inflammatory cytokines.

The cGAS-STING signaling pathway releases a variety of inflammatory factors and chemokines by inducing the production of IFNs and interacting with the NF-κB signaling pathway. This mechanism has significant implications in antiviral immunity, inflammatory responses, and a variety of diseases, including autoimmune and chronic inflammatory diseases (Figure 1).

## 3 cGAS-STING signaling pathway in chronic inflammatory lung diseases

**Figure 2:**
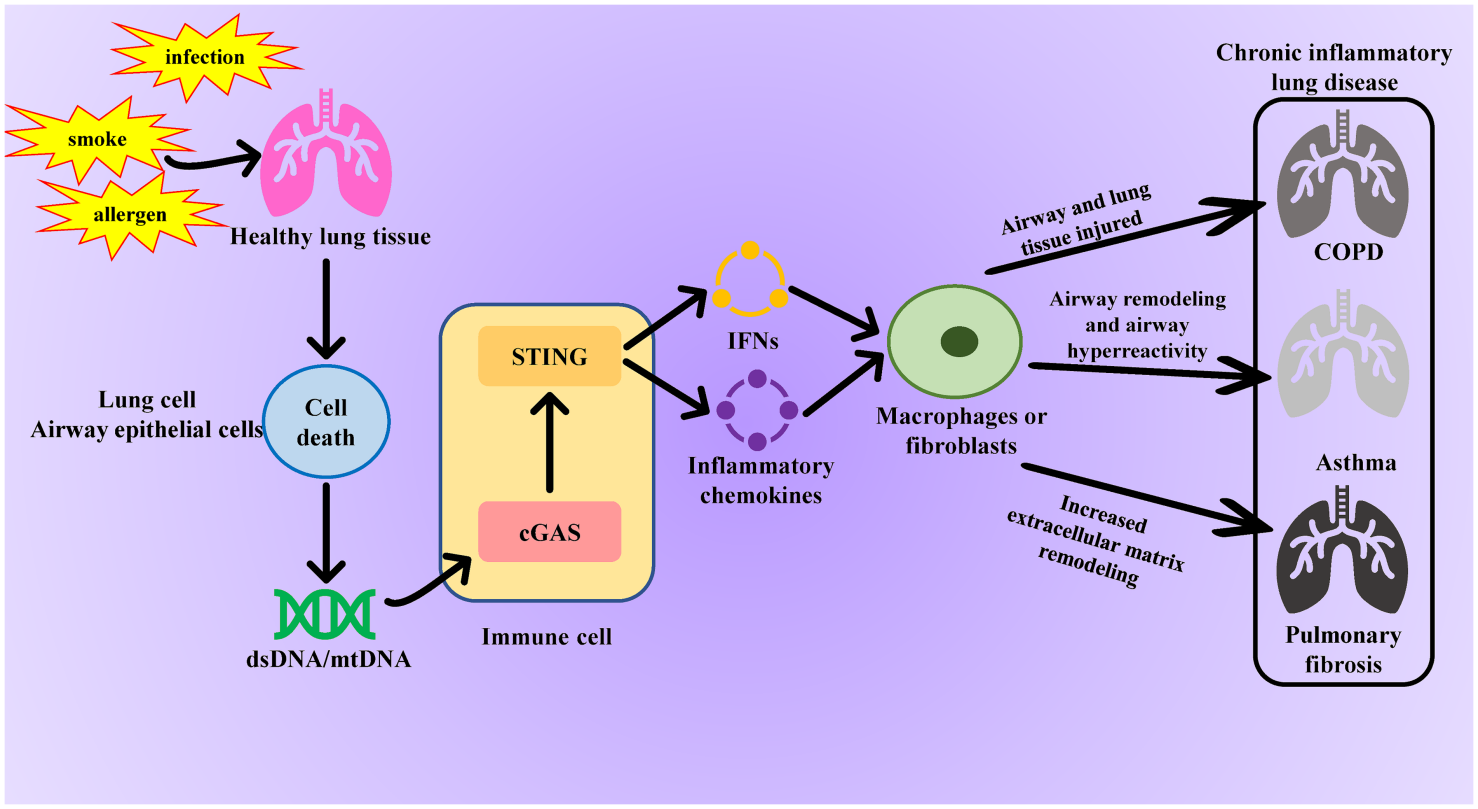
Role of the cGAS-STING pathway in chronic inflammatory lung disease. When normal lung tissue is stimulated by smoking or infection, it causes necrosis of lung cells or tracheal epithelial cells, leading to the release of dsDNA or mtDNA and activation of the cGAS-STING pathway in immune cells. The activated cGAS-STING pathway promotes fibroblast proliferation and excessive collagen deposition by releasing inflammatory factors, resulting in lung tissue fibrosis and further damage to the airways and alveoli in patients with COPD. Additionally, these inflammatory factors disrupt immune balance in asthma, exacerbating airway inflammation and allergic reactions.

The cGAS-STING signaling pathway not only plays a crucial role in pathogen infections and autoimmune diseases but also significantly contributes to the occurrence and development of many aseptic inflammatory diseases. In chronic lung diseases such as chronic obstructive pulmonary disease (COPD), asthma, and pulmonary fibrosis, continuous cellular damage and repair processes result in the release of endogenous DNA. This activates the cGAS-STING pathway, perpetuating and exacerbating chronic aseptic inflammation, which in turn leads to disease progression and deterioration (Figure 2).

### 3.1 The role of cGAS-STING in pulmonary fibrosis

Pulmonary fibrosis is an irreversible and fatal lung disease characterized by the overproduction of the extracellular matrix (ECM) and the abnormal activation of fibroblasts. Under normal circumstances, the ECM provides structural support for lung tissue. However, in pulmonary fibrosis, fibroblasts proliferate and activate abnormally, continuously producing ECM. The excessive accumulation of ECM components, such as collagen and fibronectin, leads to tissue stiffness and loss of function, ultimately resulting in severe impairment of lung function in patients (29).

Studies have shown that silica can induce lung cell death in mice during animal experiments. The double-stranded DNA (dsDNA) from the mitochondria and genome of the dead lung cells is released into the bronchoalveolar space, thereby activating the STING pathway. Additionally, increased levels of circulating dsDNA and CXCL10 have been found in the sputum of silicosis patients, with STING activation observed in their lung tissue. *In vitro* cell experiments have also demonstrated that silica induces the death of human peripheral blood mononuclear cells (PBMCs), activating the STING and type I IFN pathways (30, 31). By injecting bleomycin (BLM) into the trachea to create a mouse model of pulmonary fibrosis, researchers discovered that activation of the cGAS-STING signaling pathway promotes the expression of inflammatory factors and accelerates the aging of lung fibroblasts. They also found that inhibiting the cGAS-STING pathway with siRNA down-regulates cell cycle-related factors p16 and p21. Moreover, treatments such as Tanreqing (TRQ) and fluvoxamine effectively inhibit the release of inflammatory factors and reduce the level of pulmonary fibrosis by targeting the cGAS-STING signaling pathway (32–34).

Experimental studies and clinical observations in patients with STING-associated infantile vasculopathy have shown that individuals carrying gain-of-function mutations in TMEM173 are more likely to develop pulmonary fibrosis (35–37). Persistent activation of STING due to these mutations triggers a chronic and excessive inflammatory response. The resulting production of type I interferons and other inflammatory factors can further damage lung tissue, and the continuous inflammatory environment promotes the proliferation and activation of fibroblasts, which produce large amounts of ECM, leading to lung tissue stiffness and fibrosis (38).

However, other researchers have found that STING activation can also improve the rate of fibrosis progression. For example, Florence et al.’s study showed that STING knockout in idiopathic pulmonary fibrosis (IPF) mouse models leads to increased pulmonary fibrosis, increased lung collagen deposition, and overexpression of remodeling factors in mice (35). Therefore, further exploration of the correlation between the cGAS-STING signaling pathway and pulmonary fibrosis is crucial for developing novel therapeutic methods.

### 3.2 Role of cGAS-STING signaling pathway in asthma

Asthma is a common chronic respiratory disease characterized by airway obstruction, chronic lung inflammation, eosinophilic infiltration, excessive mucus secretion, airway remodeling, and airway hyperresponsiveness.

In a clinical study, we analyzed nasal lavage samples collected after rhinovirus (RV) vaccination in subjects with atopic asthma and healthy controls. We found that the concentration of nasal dsDNA in the upper respiratory tract increased after RV vaccination in both healthy and asthmatic subjects. Asthmatic subjects showed higher upper and lower respiratory symptom scores (RSS). Two additional clinical studies revealed that RNA sequencing results from asthma patients indicated increased expression of IFN-stimulated genes (ISG) and endoplasmic reticulum (ER) stress-related genes. Furthermore, the mtDNA copy number in the serum of asthma patients was significantly correlated with blood eosinophil count (39–42). In animal experiments, it was found that the total IgE in the serum of asthmatic mice decreased after STING knockout. Additionally, the proportion of B cells and IgE-positive B cells in alveolar lavage fluid and mediastinal lymph nodes also significantly decreased. Conversely, the addition of cGAMP increased the serum IgE level and the proportion of B cells in asthmatic mice (43). These findings suggest that activation of the cGAS-STING signaling pathway exacerbates the severity of bronchial asthma. Consequently, drugs targeting the inhibition of the cGAS-STING pathway May become new therapeutic agents for treating airway inflammation in asthma.

### 3.3 Role of cGAS-STING signaling pathway in chronic obstructive pulmonary disease

Chronic obstructive pulmonary disease (COPD) is a prevalent chronic inflammatory airway disease that poses a significant global health burden. Its pathological features include chronic lung inflammation, alveolar destruction (emphysema), and bronchiole obstruction, ultimately leading to impaired lung function (44, 45). Smoking is the primary risk factor for COPD, as harmful substances in tobacco smoke directly stimulate airway and lung tissue, triggering inflammatory responses and structural damage. Air pollution (such as particulate matter, nitrogen dioxide, ozone, etc.) can also cause and exacerbate COPD.

Oxidative stress is elevated in COPD patients, particularly during acute exacerbations. Cigarette smoke, air pollution, and biomass smoke are the main exogenous sources of oxidative stress in the lungs (46). Oxidative stress induced by cigarette smoke leads to dysregulation of mitochondrial membrane potential and dynamics, triggering the release of mitochondrial DNA (mtDNA) in cells. Studies have found that levels of 8-hydroxy-2-deoxyguanosine (a biomarker of oxidative DNA damage) are increased in the peripheral lungs of both normal smokers and COPD patients, and that plasma mitochondrial DNA levels are positively associated with COPD severity, eosinophil counts, and mortality in follow-up patients (47–50). Animal experiments have shown that the mRNA and protein expression levels of cGAS and STING are significantly increased in the lungs of mice exposed to cigarette smoke. The activation of the cGAS-STING signaling pathway then releases IFN-I, exacerbating lung injury in these mice. Additionally, activation of the cGAS-STING pathway worsens lung inflammation and tracheal remodeling following cigarette smoke exposure. Experimental results from chronic ozone exposure in mice also demonstrate that ozone can destroy airway epithelial cells, leading to the release of mtDNA and activation of the cGAS-STING signaling pathway. This induces cell death and chronic bronchial inflammation accompanied by emphysema in mice, resembling the COPD pathology induced by cigarette smoke (51, 52).

Overall, the activation of the cGAS-STING signaling pathway plays a crucial role in the onset and progression of COPD, and inhibiting this pathway is expected to offer a novel approach for COPD treatment.

## 4 Classification and characteristics of STING inhibitors

Current studies generally believe that the activation of the cGAS-STING signaling pathway leads to the release of numerous inflammatory factors (such as TNF-*α*, IL-6, IL-1β) and oxidative stress-related molecules (such as ROS). These molecules not only directly damage lung tissue but also trigger a broader inflammatory response that plays a critical role in many chronic lung diseases (53–56). Therefore, the application of STING inhibitors is expected to provide a new treatment option for these diseases (57, 58).

STING inhibitors can be classified into covalent compounds and non-covalent compounds based on how they bind to STING proteins (59). Covalent compounds form stable covalent bonds with STING proteins, permanently inhibiting their activity. Existing covalent compounds include C-176, C-178, H-151, and NO2-FAs. Among these, NO2-FAs are lipid molecules produced by the reaction of nitrite and fatty acids. They can form covalent addition reactions with specific cysteine residues (Cys88 and Cys91) on STING, causing a conformational change and preventing palmitoylation, thus blocking STING activation and transport. NO2-FAs are currently in phase II clinical trials for the treatment of pulmonary hypertension and are expected to enter clinical use in the future due to their high safety and tolerability (60, 61). The nitrofuran derivatives C-178, C-176, and H-151 inhibit STING palmitoylation by covalently binding to the Cys91 site of mouse and human STING, thereby preventing STING from binding to its agonists (such as cGAMP). The covalent modification irreversibly alters the structure of STING, blocking its polymerization process and preventing the activation of downstream signaling pathways. This significantly reduces the production of type I interferons and other inflammatory factors, thereby inhibiting inflammatory activation (62–64). These inhibitors are typically designed to be highly specific, targeting particular amino acid residues in STING proteins. The advantage of covalent compounds is their prolonged inhibition and high specificity, reducing the impact on non-target proteins. However, the design and optimization process is complex due to irreversible binding, which May cause long-term side effects or toxic reactions, and requires consideration of specific binding to the target protein.

Non-covalent STING inhibitors mainly include Compound 18 and Astin C. Compound 18 and Astin C inhibit the activation of the STING downstream signaling pathway by competitively inhibiting the binding of 2′3’-cGAMP to the STING protein and preventing STING from recruiting IRF3, respectively (65–67). Non-covalent compounds bind to STING proteins through weaker intermolecular interactions, reversibly inhibiting their activity. While non-covalent compounds are relatively better at reducing long-term side effects and toxicity through reversible binding, the downside is that the inhibition time is shorter, requiring frequent large doses. Additionally, non-specific interactions with other proteins May occur, leading to off-target effects (Table 1).

**Table 1:** Classification and characteristics of STING inhibitors.

Classification	Name	Advantage	Shortcoming
covalent inhibitors	C-176	Durability: can provide long-term suppression effect; High specificity: Well-designed covalent inhibitors can target the target protein very specifically.	Potential toxicity: May cause long-term side effects or toxic reactions; The design optimization process is complex and difficult to mass produce
	C-178		
	BB-Cl-amidine		
	H-151		
	C-170		
	C-171		
	NO2-cLA		
	9-NO2-OA		
	10-NO2-OA		
Non-covalent inhibitors	Astin C	Less toxic side effects: reversible binding can reduce long-term side effects and toxicity; Simple design: low cost, can be widely used.	Low specificity: non-specific interaction with other proteins may occur; Weak stability: Due to the weak strength of non-covalent binding, it is necessary to increase the dose and frequency of administration.
	Compound 18		
	SN-011		

## 5 Application of nano drug delivery system in STING inhibitors

In recent years, nanoplatform-based combination therapies have shown great promise in the treatment of inflammatory diseases, particularly pulmonary inflammatory diseases (68–72). Due to their small size, large surface area, and high surface activity, nanomaterials can achieve efficient drug loading and targeted delivery. Moreover, surface modifications can enhance the biocompatibility and stability of nanomaterials, thereby reducing side effects. Zhou et al. have developed an active nanomaterial encapsulating the NF-κB inhibitor MLN4924 liposomal formulation and the STING inhibitor H-151, leveraging the inflammation-targeting properties of neutrophils. By coating the nanomaterials with neutrophils, anti-inflammatory agents are effectively transported to the site of pulmonary inflammation to inhibit the NF-κB and STING inflammatory pathways, resulting in reduced cytokine production, effectively mitigating the cytokine storm, and significantly improving the therapeutic outcomes and survival rates in mice with acute pneumonia (73). However, it is regrettable that there are currently no studies on the use of nanomaterial encapsulation technology to deliver STING inhibitors for the treatment of chronic pulmonary inflammatory diseases. Therefore, extensive laboratory research, clinical trials, and data support are still needed to ensure the efficacy and safety of nanomaterial delivery systems in the treatment of chronic pulmonary inflammatory diseases. In conclusion, nanoplatform-based combination therapies offer a new approach to treating inflammatory diseases. With the continuous development and improvement of nanotechnology and advancements in STING inhibitors, this field holds promise for further innovation and application, bringing more hope to patients.

## 6 Conclusion

The innate immune system plays a crucial role in inflammatory diseases, with the cGAS-STING signaling pathway being a key component of this system. However, its excessive activation can lead to tissue damage and chronic inflammation. Animal experiments and clinical studies on pulmonary fibrosis have shown that STING activation accelerates the progression of pulmonary fibrosis by promoting the release of fibrotic factors. Regarding asthma, animal experiments have demonstrated that excessive STING activation results in airway hyperreactivity and increased mucus secretion, further exacerbating asthma symptoms. Similarly, COPD-related experiments have confirmed that overactivation of the cGAS-STING pathway worsens lung function in COPD patients. Therefore, targeting the excessive activation of the cGAS-STING pathway by combining STING inhibitors with nano-drug delivery systems May become a significant strategy for treating these chronic pulmonary inflammatory diseases.

However, considering the critical role of the cGAS-STING pathway in anti-infective immunity, a key challenge for future research will be how to inhibit pathological inflammation while preserving normal immune function. As research advances and technology progresses, targeting this pathway holds the promise of improving the quality of life and prognosis for patients with chronic pulmonary inflammatory diseases.

## Figures and Tables

**Table:** 

PRRS	pattern recognition receptors
PAMPs	pathogen-associated molecular patterns
cGAS	cyclic GMP–AMP synthase
STING	stimulator of interferon genes
dsDNA	double-stranded DNA
cGAMP	cyclic GMP-AMP
ER	Endoplasmic reticulum
TBK1	tank-binding kinase 1
IKK	inhibitor of kappa B kinas
IRF3	interferon regulatory factor 3
IFN	interferon
NF-kB	nuclear factor kappa B
TAK1	TGF-beta-activated kinase 1
SAVI	STING-associated infantile vasculopathy
RV	rhinovirus
COPD	chronic obstructive pulmonary disease
mtDNA	Mitochondrial DNA
